# Immune-related mechanisms and immunotherapy in extragonadal germ cell tumors

**DOI:** 10.3389/fimmu.2023.1145788

**Published:** 2023-04-17

**Authors:** Weigang Xiu, Jiyun Pang, Yang Hu, Huashan Shi

**Affiliations:** ^1^ 1Division of Thoracic Tumor Multimodality Treatment, Cancer Center, West China Hospital, Sichuan University, Chengdu, China; ^2^ West China School of Medicine, Sichuan University, Chengdu, China; ^3^ Department of Thoracic Surgery, West China Hospital, Sichuan University, Chengdu, China; ^4^ Department of Biotherapy, Cancer Center, West China Hospital, Sichuan University, Chengdu, China

**Keywords:** extragonadal germ cell tumor, immunohistochemistry, immunotherapy, mechanism, diagnosis

## Abstract

**Purpose of review:**

Extragonadal germ cell tumors (EGCTs) are relatively rare tumors, accounting for 1%–5% of all GCTs. In this review, we summarize the current research progress regarding the pathogenesis, diagnosis, and treatment of EGCTs from an immunology perspective.

**Recent findings:**

The histological origin of EGCTs is related to a gonadal origin, but they are located outside the gonad. They show great variation in morphology and can occur in the cranium, mediastinum, sacrococcygeal bone, and other areas. The pathogenesis of EGCTs is poorly understood, and their differential diagnosis is extensive and challenging. EGCT behavior varies greatly according to patient age, histological subtype, and clinical stage.

**Summary:**

This review provides ideas for the future application of immunology in the fight against such diseases, which is a hot topic currently.

## Introduction

Extragonadal germ cell neoplasms are rare tumors with specific molecular and pathological features. The different types of extragonadal germ cell tumors (EGCTs) include teratomas (mature or immature), seminomas, nonseminomas, and mixed GCTs (1). Compared with conventional gonadal GCTs, the histological components are basically the same, but their biological behavior, clinical characteristics, and prognosis differ greatly. Research on the origin of EGCTs has proven a relation to genetic diseases such as Klinefelter syndrome, Marfan syndrome, and Down syndrome, and there are now widely accepted theories on the origin of these tumors (2–4). Migration disorders may occur during this process, and EGCTs are formed after malignant transformation of primordial germ cells (5, 6). The incidence of EGCTs is closely related to the age of patients, with EGCTs representing up to 16% of all mediastinal neoplasms in adults and up to 19%–25% of cases in pediatric populations (<18 years). The overall incidence ranges from 1.8–3.4 per million, and females are less commonly affected than males. Teratoma is by far the most common histotype in females, representing up to 90% of all EGCTs. Teratoma, seminoma, YST and mixed tumors are quite equally represented in males. Seminoma is extremely rare before puberty and particularly in children less than 10 years of age. The most common anatomical sites if EGCTs are the sacrococcygeal region, intracranial membranes, etc., whereas seminomas are extremely rare (7, 8) (**Table 1**). The incidence of mixed teratoma is higher in the elderly. In adults, the location of EGCTs varies according to sex. In a statistical study of gonadal GCTs and EGCTs by Stang et al. (9), the most common extra-gonadal sites were the mediastinum, pineal gland, retroperitoneum, and brain in men, and the placenta, pelvis, uterus, and brain in women. In addition, isolated cases have been reported in the kidney, paranasal sinuses, and vulva (10, 11). Moreover, it remains unclear whether carcinoma in situ, intraepithelial seminoma, testicular seminoma, etc., are the causes or precursors of tumor metastasis and extragonadal malignancy (12). Because of the rarity of the disease and poor categorization, novel therapeutic approaches are urgently needed for this tumor type.

**Table 1:** Clinical features of EGCTs (1, 7, 8).

Age at diagnosis	Histopathology	Anatomical locations	Genetic syndrome
20-35 years	Seminoma	Mediastinum	Klinefelter
	Nonseminoma	Pineal and suprasellar regions	Hematologic
		Sacrococcyx (infants and young children only)	Malignancies

EGCTs, extragonadal germ cell tumors.

## Immunological pathogenesis

It is generally accepted that EGCTs, like primary gonadal tumors, follow the principle of development from primordial germ cells. Migration from the inner wall of the yolk sac to the midline genital crest is abnormal, and primordial germ cells remain outside the gonad and develop into an EGCT. Therefore, deletion of the long arm of chromosome 12 and one or more copies of the short arm of the same chromosome are found in various primary adenocarcinomas and EGCTs (13, 14). However, one study compared the clonal cytogenetic changes of primary gonadal tumors and mediastinal germ cell tumors and found no significant difference in chromosomal characteristics, such that extragonadal tumors may originate from the gonads (15).

From the perspective of immunology, the occurrence of EGCTs is closely related to immune escape. The concept of immune surveillance was first proposed by Ehrlich in 1909. It is believed that the host immune system constantly recognizes and eradicates evolving tumors before the clinical manifestations of tumors appear. With suppression of the immune system, cancer will occur frequently (16). Fifty years later, Thomas (17) proposed that low expression of tumor cell antigens or impaired cellular immune function are important factors for the occurrence of tumors, based on a study of the evolutionary mechanism of body cellular immunity. Later, Burnet refined the concept of immune surveillance in 1970, suggesting that genetic changes leading to malignancies are common in somatic cells; that the immune system is responsible for eliminating or inactivating these potentially dangerous mutated cells; and that tumors occur when immune surveillance is inadequate to effectively eliminate “alien” components or mutated cells (18). However, in 2001, Frances Balkwill and colleagues proposed a concept completely contrary to the cancer immune surveillance hypothesis, suggesting that inflammatory immune cells and cytokines in tumors may promote rather than inhibit tumor growth (19). Although controversial, research has shown that severe primary immunodeficiency is associated with an increased risk of malignancy, indirectly demonstrating that precancerous lesions can be eradicated by internal immunity and that the immune system can play a protective role against cancer (20). Non-virus-related tumors such as EGCTs are not among the cancers frequently seen in immunodeficient individuals, but they are still associated with impaired immune system surveillance. For example, when HIV-1 infects and kills CD4+ T cells, leading to acquired immunodeficiency, cancers such as EGCTs that are not associated with oncogenic viruses are also more common. Especially in cases of organ transplantation, EGCTs are more likely to occur if immune deficiency is found due to pharmacological immunosuppression (21, 22). In conclusion, with the real-time monitoring and timely clearance of abnormal cells by the human immune system, the occurrence of tumors, including EGCTs, can be avoided. However, when immune dysfunction and immune deficiency occur, the incidence of EGCTs is significantly increased.

Several immune escape mechanisms have been identified, and one of the key mechanisms is the loss of immunogenicity and antigenicity by tumor cells. Tumor cells exert their immunosuppressive effects by antagonizing, blocking and suppressing the body’s immune response through their structural and non-structural products (23). As mentioned above, under normal circumstances, the immune system can resist tumorigenesis through natural and acquired immunity, which affords timely recognition and elimination of these diseased germ cells to resist the occurrence and development of EGCTs. However, malignant cells escape immune surveillance through various mechanisms and proliferate rapidly *in vivo* to form GCTs. Understanding the immune escape mechanism of EGCTs can support the development of strategies to reverse the corresponding immune escape mechanism as well as new immunotherapy regimens and new approaches for EGCT treatment.

## Formation of immunosuppressive microenvironment

Tumor cells can form immunosuppressive microenvironments in a variety of ways to achieve immune escape. First, tumor cells can secrete immunosuppressive cytokines or metabolic factors, such as transforming growth factor-β (TGF-β), interleukin-6 (IL-6), IL-10 and prostaglandin (PGE2), in an autocrine or paracrine manner to inhibit the killing of tumor cells (24, 25). Studies have shown that plasma levels of IL-6, IL-10 and other contents in GCTs and embryonic tumors of the central nervous system are increased and can be improved by corresponding treatment (26). Vivian et al. (27) also found that podoplanin is upregulated in GCTs, and its content is positively correlated with various immunosuppressive cytokines. Therefore, abnormally high levels of immunosuppressive factors produced by cancer cells, which suppress immunity, are a prerequisite for tumor growth (28). At the same time, GCT cells can also induce host production of immunosuppressive cells, which play a negative regulatory role in the body’s anti-tumor immune response. Studies have demonstrated the presence of regulatory T cells (Tregs) in the blood and tumor tissues of tumor patients that can inhibit the body’s anti-tumor immune response. Tregs can be CD4+ and CD8+T cells and are able to inhibit the proliferation and activation of effector T cells and to inhibit the secretion of T helper cell (Th) cytokines to inhibit the body’s anti-tumor immune response. For example, Retana et al. (29) found that both pediatric malignant extracranial GCTs (meGCTs) and adjacent tumor subtypes belonging to mixed meGCTs are permeated by CD4+ and CD8+T cells in various ways. Clinically, Tregs have been shown to be closely associated with tumor staging and prognosis, and removal of Tregs can evoke effective anti-tumor immunity by eliminating the immune response to syngeneic tumors. In addition, myeloid-derived suppressor cells (MDSCs) are also widely present in peripheral blood and tumor tissues of EGCT patients (30). These cells include immature macrophages, granulocytes, dendritic cells (DCs), and so on. After reaching the periphery, these cells are further activated and, as a result, can express a variety of pro-angiogenic factor and, inhibit T cells, natural killer (NK) cells and other immune responses, in order to participate in the suppression of anti-tumor immunity.

**Figure 1:**
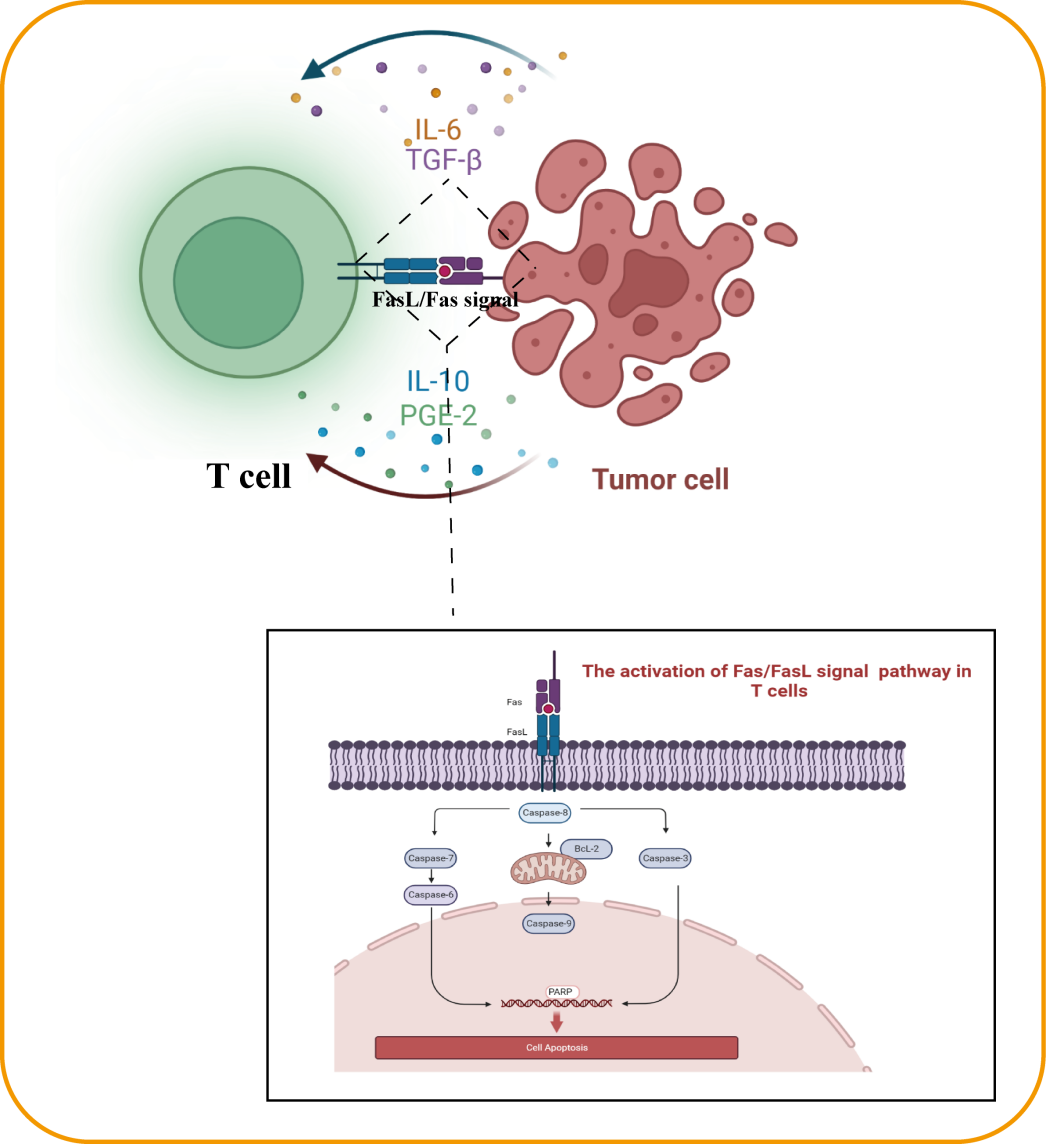
The anergy in EGCT infiltrating lymphocyte.

Fas protein, a member of the TNF receptor family, binds to the Fas ligand (FasL) and induces apoptosis in cells expressing Fas protein (31). Due to the high expression of FasL in surface cells of EGCTs, GCT cells can mediate the apoptosis of immune effector cells through the FasL/Fas pathway, which weakens the effectiveness of the body’s immune response. Studies have shown that production of FasL by GCT cells may lead to early development of GCTs by inducing apoptosis of FAS-positive–, Fas-associated phosphatase-1 (FAP-1)–negative tumor-infiltrating lymphocytes (**Figure 1**) (32).

## Immune cell recognition and activation

EGCT cells are derived from the original germ cells of the body, and the antigens expressed by EGCT cells differ very little from the body’s normal proteins. Therefore, the immunogenicity of these cells is very weak, and the immune response of the body is not strong enough. The immunogenicity of some tumor cells is relatively strong though, but as these cells are cleared by the immune response, the overall trend is the growth of cells with continually weaker immunogenicity. This allows EGCT cells to successfully escape recognition by immune cells.

EGCT cells may also have decreased or absent major histocompatibility antigen-I (MHC-I) expression. DC-mediated presentation of MHC-I class/peptide complexes is a critical first step in initiating cytotoxic T lymphocyte (CTL) responses (33). When MHC-I expression is reduced, CTL activation and killing of tumor cells cannot be induced. Human leukocyte antigen G (HLA-G) is expressed by trophoblast cells outside placental villi and can also mediate immune escape. Abnormal expression of HLA-G by tumor cells can inhibit killing of tumor cells by T cells and antigen-presenting cells (34, 35). Research has shown that the human HLA-G–positive choriocarcinoma cell line JEG-3 increases the expression of HLA-G+ CD4+ T cells and mediates maternal–fetal immune escape during pregnancy (36). DC antigen presentation dysfunction may occur in EGCT patients. DCs can efficiently absorb, process and present antigens, and thereby significantly stimulate the activation of initial T cells, serving as the initiator of the body’s T-cell immune response. Due to the disruption of tumor antigen presentation, EGCT cells are not recognized by immune cells.

Furthermore, EGCT cells can show abnormal signals of costimulatory and adhesion molecules. Activation of two signals simultaneously is needed to fully activate T cells, including the peptide/major histocompatibility complex II with T-cell receptors and additional signals transferred by stimulating molecules (37). However, tumor cells express positive costimulatory molecules, such as CD80 and CD86, at reduced levels that are insufficient to provide an effective second signal for T-cell activation, and thus, cannot effectively induce the anti-tumor immune response, generating immune tolerance instead.

## Immunogenicity of tumor cells

The anomalous expression of tumor-associated antigens along with immunosuppressive factors and immune cell dysfunction greatly interfere with the immune recognition process, allowing tumor cells to further escape clearance by the immune system and killing by drugs. For example, the treatment of EGCT mainly relies on cisplatin chemotherapy, with a high cure rate. However, some patients show resistance to cisplatin, which greatly increases the difficulty of treatment. Studies have found that drug-resistant GCTs are associated with mutations in the TP53 gene, which inactivate its function and thereby prevent apoptotic responses after contact with immune cells (38). Mutations in tumor cells can also promote their own survival and growth. Yue et al. (39) and De et al. (40) reported concurrent germline *SDHA* and somatic *KIT* mutations in pediatric central nervous system GCTs and *SDHBp.L157W* gene mutations in mediastinal GCTs, and speculated that such mutations could promote tumor angiogenesis and cell proliferation by activating the pseudo-hypoxia pathway.

## Immunological diagnosis

### Immunohistochemistry

EGCTs often present only at advanced stages when tumor-related symptoms finally occur, but they also can be incidental findings during diagnostic or other therapeutic interventions. Meanwhile, the clinical presentation of EGCTs varies widely, which adds to the difficulty of diagnosis (12). Therefore, the rational application of immunohistochemistry (IHC) analyses is very important. Notably, Spalt-like transcription factor 4 (SALL4) and placental alkaline phosphatase (PLAP) are common markers of malignant GCTs. However, the use of PLAP is limited due to its discrepant sensitivities and expression patterns in different tumors, and sometimes not obvious staining in the cytoplasm or cell membrane (41, 42). SALL4 is an important transcription factor associated with embryonic cell pluripotency (43). Its advantage is that it has good sensitivity and specificity for tumors, with positive staining observed in the nucleus, accurate localization, and obvious characteristics (44). SALL4 is expressed in germ cells and is an excellent marker for malignant GCTs, because most of the tumor cells stain strongly and uniformly positive. For example, the sensitivity of SALL4 for embryonal carcinoma and yolk sac tumors is almost 100%. The expression rate of SALL4 in immature teratomas is about 75%, whereas it is not expressed in mature teratomas (45–47). These results also apply to primary EGCTs of the mediastinum and central nervous system. In general, IHC staining for SALL4 is a powerful tool for the diagnosis of EGCTs when used in combination with GCT markers.

No specific marker has been discovered for extragonadal yolk sac tumors, but alpha fetoprotein (AFP) is a relatively characteristic marker for the epithelial component of yolk sac tumors in general. AFP was one of the first protein tumor markers identified and is widely used for tumor screening, diagnosis, and prognosis (48). For the diagnosis of yolk sac tumors, however, the overall sensitivity of AFP staining is not high. Still AFP is highly specific for yolk sac tumors and is not expressed or only occasionally expressed in embryonic tumors, teratomas and other tumor types. Therefore, AFP staining remains a good protocol for identifying yolk sac tumors. To diagnose yolk sac tumor with high accuracy though, comprehensive evaluation with the assistance of other markers is needed. Glypican-3 is an effective supplement. It is a cell-surface heparan sulfate proteoglycan that regulates cell growth during fetal development (49, 50). Importantly, it is normally not expressed in healthy adult tissues and exists as a carcinoembryonic antigen. It is also specific for yolk sac tumors, which helps to avoid confusion even when other tumors are positive (e.g., choriocarcinoma). Notably, it is important to distinguish an EGCT from hepatocellular carcinoma, hepatocellular carcinoma cells can also express AFP and glypican-3. SALL4 is another marker that can aid the diagnosis of yolk sac tumors (51). ZBTB16 also shows high sensitivity and specificity for yolk sac tumors and was found to be expressed in 91.6% of extra-gonadal and metastatic yolk sac tumors (52). Combined application of the above markers can be used for the effective detection of yolk sac tumors.

Embryonal carcinoma is usually present as a large mass at diagnosis, with features of infiltration of surrounding tissue. The symptoms and signs of embryonal carcinoma vary depending on the location (53). For extragonadal embryonal carcinoma, IHC diagnosis also requires a combination of multiple markers. First, SALL4 is a widely used marker for these tumors (54). A positive result from SALL4 staining is used as a preliminary determination step. In addition, octamer-binding transcription factor 4 (OCT4) can be diffusely expressed in the nucleus of extragonadal embryonic tumors and is one of the essential stem cell factors in embryogenesis and pluripotency (55). NANOG (Nanog homeobox) is mainly expressed during embryonic development and can inhibit cell apoptosis, leading to drug resistance. It is generally not used as a first-line marker but can play a role in the diagnosis of extragonadal embryonal carcinoma, because it is found in the nucleus and not expressed yolk sac tumors, teratomas, or choriocarcinoma (4). Cluster of differentiation 30 (CD30) has high specificity for extragonadal embryonic tumors, but its sensitivity is lower than that of other markers (56). Moreover, its sensitivity may be further reduced after chemotherapy. For example, research has proven that OCT4 is a useful diagnostic marker to identify metastatic embryonal carcinomas after chemotherapy, with a better sensitivity than CD30 (57). SOX2 is a transcription factor involved in cancer progression that promotes cancer cell migration, invasion, and proliferation (58). It is also one of the markers used for the diagnosis of extragonadal embryonal carcinoma, but its use alone may lead to misdiagnosis (59). Overall, when tumor cells simultaneously express CD30, SOX2, OCT4 and SALL4, the diagnosis of extragonadal embryonal carcinoma is almost certain.

Most teratoma diagnoses do not require the use of IHC. However, if necessary, IHC can be used to ensure the accuracy of a teratoma diagnosis (4). At present, markers for these tumors are still being tested and discovered. For example, one study reported that terminal deoxynucleotide transferase (TdT) can be used as a new IHC marker for the diagnosis of various extragonadal GCTs with a high positive rate in seminomas (99%, 107/108), ECs (100%, 15/15), and extragonadal germinomas (100%, 11/11) and absence in YSTs (0/38) and teratomas (0/19) (60). More clinical trials targeting various types of EGCTs are expected in the future. With the expansion of the database and the discovery of IHC markers, methods for the accurate diagnosis and effective treatment of EGCTs can be significantly improved.

For IHC diagnosis of choriocarcinoma, the conventional method is staining for both SALL4 and human chorionic gonadotropin (HCG). HCG, which is produced primarily by differentiated syncytic cells, plays a specific role in promoting uterine endothelial angiogenesis, maintaining uterine muscle quiescence, and promoting immune regulation at the maternal–fetal interface (61). It is generally not expressed in other types of EGCTs, and thus, it can be used to diagnose extragonadal choriocarcinoma and is the most sensitive marker of choriocarcinoma. At the same time, SALL4 was expressed in 100% of choriocarcinomas, and it was not detected in any placental site trophoblastic tumors and epithelioid trophoblastic tumors, making it a distinguishing marker for choriocarcinomas (62). Glypican-3 and inhibin also are expressed in choriocarcinoma and can be used as diagnostic aids (63, 64). Notably, however, choriocarcinoma is sometimes a component of mixed GCTs, and therefore, multiple markers should be used in combination for analysis.

### Serum tumor markers

The detection of serum tumor markers is a simple, non-invasive testing method that is easily accepted by patients. The method involves the measurement of specific biochemical serum tumor markers (STM) and has high clinical application value (65, 66). With the appearance of tumors, carbohydrate antigens, hormones, receptors, enzymes, oncogenes and tumor suppressor genes are produced by tumor cells and secreted into serum. Detection of these molecules can reflect the presence of tumors to a certain extent, and then testing for chronic inflammation, benign tissue hyperplasia and infection can be carried out.

AFP is one of the commonly used STMs in EGCT patients. In non-hormone–related EGCTs, AFP content is often significantly increased, and the increase is proportional to disease stage, allowing this STM to be used as a reference factor for diagnosis and staging. A stable increase in serum AFP is seen in pure yolk sac tumors and GCTs containing a yolk sac tumor (67). However, some EGCTs, such as seminomas, do not exhibit an increase in AFP (68). HCG is also a commonly used type of STM. In choriocarcinoma, the serum HCG level is higher in mixed GCTs with choriocarcinoma as compared with pure choriocarcinoma. The serum level of HCG in seminoma cases is also increased and has been shown to be associated with multinucleated trophoblastic giant cells (41). Lactate dehydrogenase (LDH) is also a common STMs. It is characterized by poor specificity, with an increase of around 50% for various histological types (4). Von Eyben et al. (69) demonstrated that serum LDH may reach higher levels when a relevant gain of chromosome 12p is present, and the LDHB gene located on 12p. Thus, it can be used to monitor risk in patients with nonseminomatous GCTs. At present, this method has only been described in testicular germ cell tumors, but it is of reference value for EGCTs. On the whole, clinical research regarding these common STMs is relatively mature, but the results of individual studies are not completely reliable due to the influence of patient characteristics and clinical conditions. Novel biomarkers with greater sensitivity and specificity are needed, and recent research has continued to identify new serum markers. For example, microRNAs (miRNAs) are receiving considerable attention. Syring et al. (70) quantified miRNA levels by quantitative real-time polymerase chain reaction and found that the levels of miR-302a-3p, 371a-3p, 372-3p and 373-3p were significantly increased in GCT patients, and these mRNAs together outperform HCG and AFP testing in terms of sensitivity (84.7%) and specificity (99%). Additionally, these serum miRNA levels decreased postoperatively, indicating tumor specific release. Spiekermann et al. (71) tested the serum level of miR-371a-3p and found that it was higher in patients with GCTs and returned to normal rapidly after treatment. The serum level of miR-371a-3p also correlated with tumor volume and other indicators, proving this miRNA to be a promising new biomarker. While these studies have demonstrated the broad potential of miRNAs as STMs, current studies are mostly limited to testicular GCTs, and the research regarding markers for EGCTs is limited. Large-scale clinical studies are needed to identify improved serum markers, including miRNAs, for EGCTs and to demonstrate their effectiveness.

## Immunotherapy

Overall, GCTs are sensitive to chemotherapy and radiotherapy, and the cure rate with these treatments is high. Therefore, chemoradiotherapy is the main treatment for GCTs, and surgical resection is used for mature teratomas (72). Studies have shown that the prognosis of EGCTs is associated with the histological type of the tumor and the expression of tumor markers (AFP and β-HCG). For example, long-term progression-free survival (PFS) is achieved in roughly 90% of mediastinal spermatogonia EGCT patients treated with chemotherapy (73). However, in another study by Bokemeyer et al. (74), the 5-year survival rate among patients with retroperitoneal nonseminoma EGCTs was only 62% and that for patients with mediastinal EGCTs was only 45%. A randomized phase III trial (73) suggested that tumor marker AFP and β-HCG response following one cycle of bleomycin, etoposide, and platinum (BEP) in men with poor-risk disease may have important prognostic value. In that study, 263 men with NSGCTs were treated with BEP using a risk-stratified approach driven by tumor marker response. Patients with an insufficient decline in tumor marker expression after one cycle of BEP were randomly assigned to either dose-dense or standard-dose BEP for the remainder of their treatment. Among this cohort, patients treated with dose-dense BEP experienced a higher 3-year PFS (59% vs 48%) than those who continued with standard BEP.

It is clear that new breakthroughs are needed in the treatment of EGCTs. In addition, the prognosis of refractory or multiplicative recurrent germ cell carcinoma is not optimal, and thus, breakthroughs in immunotherapy are needed to overcome the current difficulties.

**Figure 2:**
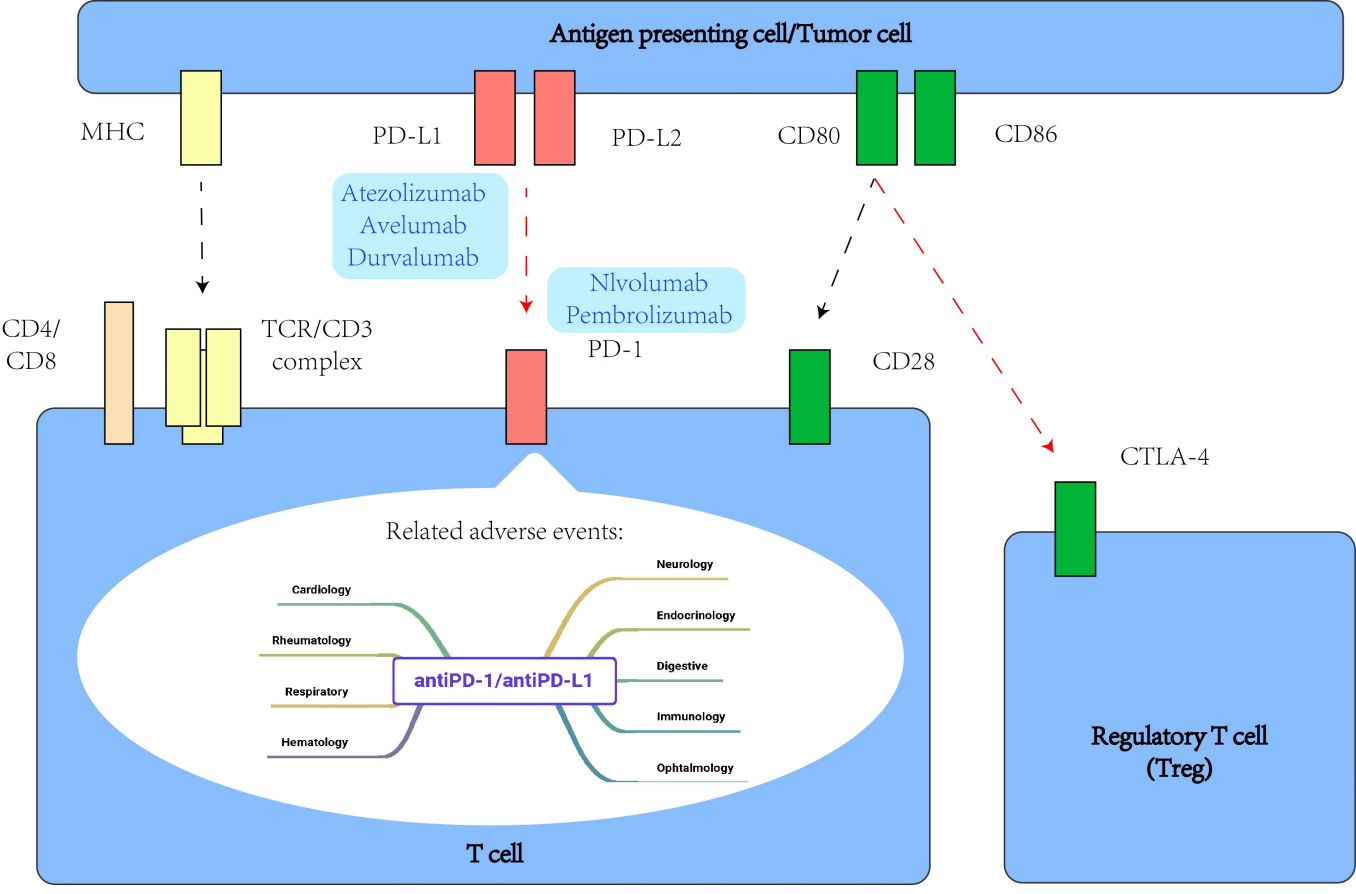
Some intracellular signaling events related to PD-1 and CTLA-4 during T cell activation, and main adverse events related to antiPD-1/antiPD-L1 agents.

Among tumor immunotherapies, tumor immune checkpoint inhibitors are the most mature and widely used in clinical research, which has great significance for the advancement of EGCT treatment (75, 76). PD-1 is a member of the T cell regulatory family, is mainly expressed in B lymphocytes and T lymphocytes, but also in NK cells, DCs, etc., and is an important negative immune regulator (77). PD-L1 is one of its ligands and is expressed in a variety of cells such as antigen-presenting cells and malignant tumor cells (78, 79). The interaction of PD-1 with PD-L1 is an important mechanism by which cancer cells inhibit anti-tumor immunity in the tumor microenvironment, resulting in immune escape of tumor cells and affecting immune homeostasis (80). Immunohistochemical analysis of PD-1 and PD-L1 in central nervous system GCT was performed by Woods et al (81), who reported that 22 germinomas (79%) were positive for PD-L1 expression and 13 NGGCTs (57%) were positive for PD-L1. Their results suggest that immune checkpoint inhibitors may be effective in treating intracranial GCTs. When binding between PD-1 and PD-L1 or PD-L2 is inhibited, non-specific reactivation of T cells can lead to an enhanced immune response and anti-tumor effect. To date, a large number of studies have demonstrated the theoretical feasibility of using immune checkpoint inhibitors to treat EGCTs. Chovanec et al. (82) analyzed the immunoinflammatory index according to platelet count, neutrophil count, and number of tumor-infiltrating lymphocytes. At the same time, many clinical studies in patients with a variety of GCTs have tested various antibody treatments such as tislelizumab and pembrolizumab, with some patients achieving remission and showing favorable clinical outcomes, while others had to stop treatment due to poor efficacy or clinically insignificant benefit (83–88). A Phase II study of pembrolizumab, an anti-PD-L1 monoclonal antibody, did not show any clinical response among the first 12 patients enrolled (NCT02499952) (89). Another Phase II study reported the effectiveness of avelumab in multiple relapsed/refractory GCT, with efficacy (12-week PFS) as a primary outcome (NCT03403777) (90). Furthermore, a two-arm, Phase II study is assessing the combination of durvalumab and tremelimumab in advanced GCT in terms of effectiveness and safety (NCT03081923) (91)(**Table 2**). From the results of these various studies, it can be surmised that tumor immune checkpoint inhibitors are generally safe and well tolerated. Antibodies against immunomodulators CTLA4 and PD-L1/PD-1 have shown some success in clinical application at times, but their effects on tumor treatment are not sufficiently stable, with the target effect often not achieved. Meanwhile, EGCTs have not been found to show better responses to immunotherapy than GCTs. As a typical location of GCTs, the testis is considered a privileged immunological site because of its immune system’s weak response to antigens and special immune environment, which prevents the germ cells from being subjected to autoimmune attack (**Figure 2**) (92).

**Table 2:** Trials evaluating the effectiveness of immune checkpoint inhibitors in GCTs (89–91).

Trial	Phase	Drugs	Estimated enrollment (no. of participants)	Outcomes	Ref.
NCT02499952	II	Pembrolizumab	12	No PR or CR cases. 50% of cases died of disease progression	(89)
NCT03403777	II	Avelumab	8	Median PFS of 1.4 months and OS of 2.7 months.	(90)
NCT03081923	II	Durvalumab tremelimumab	22	One PR case was observed in the durvalumab plus tremelimumab group.	(91)

GCTs, germ cell tumors; PR, partial response; CR, complete response; PFS, progression-free survival; OS, overall survival.

Immunotherapies that target tumors have been developed, such as adoptive cell therapy (ACT). Immune cells such as T cells in peripheral blood of patients are genetically modified or activated *in vitro* to express chimeric antigen receptor (CAR) or T-cell receptor (TCR) to improve cellular immune function (93, 94). The ability of genetically engineered lymphocytes to express conventional T-cell or chimeric antigen receptors has expanded their use, and there is also an active effort to identify and develop specific antitumor T cells with optimal functional properties (95, 96). However, this approach has limitations, such as more severe side effects, and is mostly used in the treatment of hematological tumors, with many challenges remaining to be overcome for their application in the treatment of solid tumors like EGCTs (97). Vaccination is also a treatment method that has attracted attention in recent years. This approach is divided into preventive vaccines and therapeutic vaccines (such as protein and peptide vaccines, DNA vaccines and recombinant vector vaccines). However, researchers have found it difficult to induce effective antigen-specific cellular immunity, which is still in the experimental stage, and cancer vaccine technology is still mainly applied to cervical carcinoma, with research regarding EGCTs still lacking (98).

When properly combined with other reagents and therapies, immunotherapy can also improve patient outcomes. For example, deep hypomethylation in seminoma correlates with CD8+ cell abundance, and the combined use of immunotherapy and hypomethylation reagents results in a higher tumor immunogenic phenotype (99). Wang et al. (83) described the effectiveness of adding immunotherapy to a conventional chemotherapy regimen for patients with extra-gonadal yolk sac tumors for reducing the recurrence rate and improving prognosis. Overall, immunotherapy has revolutionized cancer treatment and revitalized the field of tumor immunology, with clinical trials demonstrating its effectiveness for a variety of cancer types (100). However, for EGCTs, the efficacy of immunotherapy remains inconsistent, likely due to the complexity of the mechanism and heterogeneity of the tumor immune environment. It is hoped that future research will identify more drugs targeting various immune tolerance mechanisms and as well as effect treatment combinations to provide more better treatment options and improved prognosis for EGCTs (101).

## Conclusion

Extragonadal germ cell tumors are relatively rare and histologically similar to gonadal germ cell neoplasms, but with unique clinical and biological features and poor prognosis. With recent research discoveries, the immunological pathogenesis of these tumors has become increasingly clear, and the immunodiagnostic methods more mature, which has greatly improved the accuracy and efficiency of diagnosis. Moreover, although the current research results are not sufficiently comprehensive and stable, and large-scale safe application of new therapies will require considerable further research, new immune markers and immunotherapy methods are expected to be identified according to the relevant immune escape mechanism. Such progress will improve treatment efficacy and prognosis. However, at the same time, most of the current research on GCTs is still focused on tumors of the gonad, especially the testis, and more research on EGCTs, especially at unusual sites, is needed for clinical improvements in treatments and prognosis prediction for these patients.

## Author contributions

HS and YH designed the study. WX conducted the experiments and analyzed the data. WX and JP wrote the manuscript. All authors contributed to the article and approved the submitted version.
